# Shared Binding Properties Between a Therapeutic Antibody and Anti-Transthyretin Antibodies From Transthyretin Cardiac Amyloidosis Patients: Translational Implications for Future Clinical Trials

**DOI:** 10.31083/RCM48514

**Published:** 2026-03-09

**Authors:** Jacob George, Clara Benaim, Michael Fassler

**Affiliations:** ^1^Cognyxx Pharmaceuticals, 6901648 Tel Aviv, Israel; ^2^Heart Center, Kaplan Medical Center, 76100 Rehovot, Israel; ^3^Hebrew University, 9190501 Jerusalem, Israel

**Keywords:** transthyretin cardiac amyloidosis, monoclonal antibody, immunotherapy

## Abstract

**Background:**

Transthyretin cardiac amyloidosis (ATTR-CA) results from the extracellular deposition of misfolded transthyretin (mis-TTR) and promotes progressive cardiac dysfunction. Current therapies, such as stabilizers and silencers, reduce further fibril accumulation but fail to clear existing deposits. Monoclonal antibodies (mAbs) targeting mis-TTR have emerged as promising disease-modifying agents, supported by recent observations of circulating anti-TTR antibodies in patients who exhibited spontaneous clinical improvement.

**Methods:**

This study aimed to purify natural anti-TTR antibodies from two ATTR-CA patients and compare the respective binding properties to those of a previously described therapeutic anti-TTR mAb fragment (Ab-A F(ab')_2_). Statistical significance was determined using Student's *t*-test.

**Results:**

Both natural antibodies and the Ab-A F(ab')_2_ demonstrated high-affinity binding to misfolded TTR (n = 3), while the competition assays revealed dose-dependent inhibition, indicating shared epitope recognition.

**Conclusions:**

These findings provide translational evidence that therapeutic anti-TTR mAbs may mimic naturally protective antibodies, suggesting that these antibodies could promote amyloid clearance and deliver true disease-modifying benefits in ATTR-CA.

## 1. Introduction

Transthyretin cardiac amyloidosis (ATTR-CA) is an indolent degenerative disease that manifests clinically after significant amyloid burden has already accumulated in the extracellular space. Misfolding of native tetrameric TTR forms the nidus from which amyloid accumulates, leading to the characteristic progression from impaired cardiac diastolic function to subsequent systolic dysfunction [1]. Subsequently, advances in understanding disease progression mechanisms and in developing improved diagnostic tools have led to the active pursuit of a disease-modifying drug, the current approval of two stabilizers, and the anticipated approval of an siRNA therapy [2, 3, 4].

However, recent data suggest that the size effect of Tafamidis, approved in 2018, is at best moderate, and a significant impact on mortality is not evident [5]. Meanwhile, the mild-to-moderate effect of stabilizers/silencers is predominantly attributed to the ability of the stabilizer to reduce ongoing accumulation rather than to dispose of pre-existing, already heavily deposited amyloid, as evidenced by initial clinical signs in patients. Along these lines, therapeutic monoclonal antibodies (mAbs) to TTR in clinical development may be disease modifiers, as these antibodies target and debulk resident amyloid deposits [6, 7, 8].

Recently, Fontana *et al*. [9] identified natural polyclonal anti-TTR antibodies in patients with ATTR-CA that experienced spontaneous clinical improvement. This striking finding was supported by our subsequent observation, which reinforced the findings of Fontana *et al*. [9] and also demonstrated functional amyloid-debulking properties of circulating polyclonal antibodies [10]. Thus, we conducted this comparative study to assess whether natural anti-TTR antibody-associated clinical regression can predict the future success of therapeutic mAbs in clinical development.

Our previously described therapeutic monoclonal anti-TTR antibody was found to compete with circulating patient-derived anti-TTR antibodies for binding to mis-TTR [8]. This finding suggests that mAbs may possess protective and binding characteristics similar to those of natural anti-TTR antibodies.

## 2. Materials and Methods

Natural anti-TTR antibodies were purified from two previously described male patients with ATTR-CA and compared with those from a control patient with heart failure in whom ATTR-CA was excluded. Binding specificity was assessed by enzyme-linked immunosorbent assay (ELISA) using misfolded TTR protein (AlexoTech, Umea, Sweden) as the capture antigen. To evaluate any overlap between the epitope and therapeutic monoclonal antibody, a bivalently antigen-binding fragment (Ab-A F(ab’)_2_) was generated by pepsin (Pepsin A-LS003319, Worthington Biochemical, Lakewood, NJ, USA) digestion of the mouse IgG1 anti-TTR antibody, followed by purification. This fragment was employed to eliminate Fc-mediated non-specific interactions with the secondary antibody (peroxidase anti-human-109-035-088, Jackson ImmunoResearch, West Grove, PA, USA) and to perform competitive binding assays. Binding curves for Ab-A F(ab’)_2_ and the patient-derived natural anti-TTR antibodies were generated to calculate the half-maximal effective concentrations (EC_50_). Increasing concentrations of Ab-A F(ab’)_2_ were then tested for competitive inhibition of natural antibody binding to mis-TTR.

## 3. Results

**Fig. 1.**
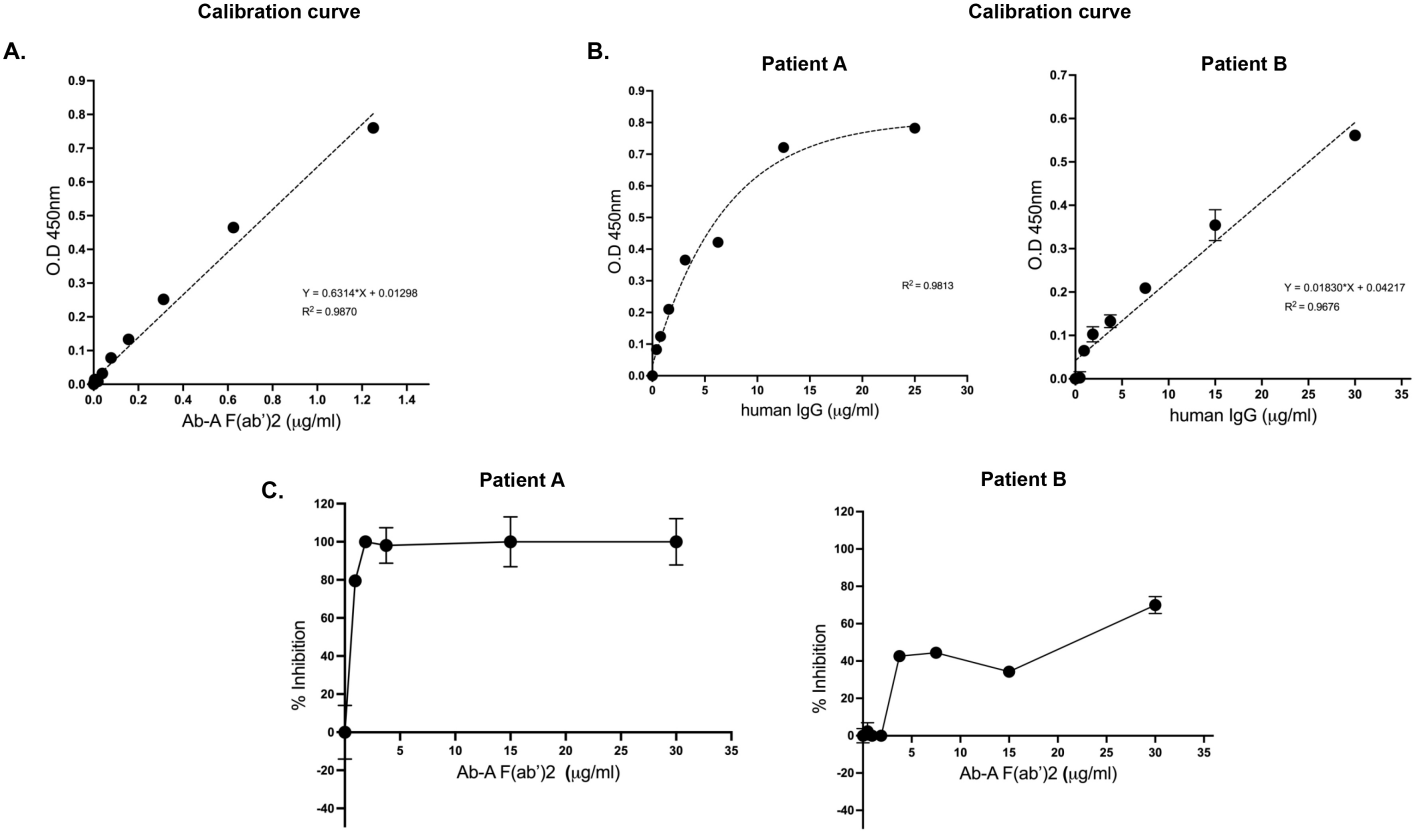
**Binding properties and competitive inhibition of natural human anti-TTR antibodies and therapeutic Ab-A**. (A) Binding curve of Ab-A F(ab’)_2_ to mis-TTR. (B) Binding curve of natural human anti-TTR antibodies to mis-TTR purified from patients A and B for EC_50_ assessment. (C) Binding of purified natural human anti-TTR antibodies from ATTR-CA patients to mis-TTR and competitive inhibition of mouse Ab-A F(ab’)_2_ to the mis-TTR. The background was reduced by the unspecific binding of ATTR-CA-purified antibodies from a control patient. Samples were measured in triplicate. TTR, transthyretin; ATTR-CA, transthyretin cardiac amyloidosis; EC_50_, effective concentrations.

Ab-A F(ab’)_2_ demonstrated dose-dependent binding to mis-TTR, confirming specific recognition of aggregated TTR (Fig. 1A). Purified polyclonal anti-TTR antibodies from both patients bound mis-TTR with measurable EC_50_ values, establishing a high-affinity interaction (Fig. 1B). Control antibodies from non-ATTR patients showed no specific binding. In the competition assays, Ab-A F(ab’)_2_ inhibited the binding of natural anti-TTR antibodies in a concentration-dependent manner (Fig. 1C), demonstrating epitope overlap. Both patient-derived antibody preparations showed consistent competition profiles, while control antibodies exhibited no specific binding. These results indicate that the therapeutic mAb shares binding properties with naturally occurring protective anti-TTR antibodies from ATTR-CA patients.

## 4. Discussion

Notably, mAbs against TTR are in clinical development for the treatment of ATTR-CA; however, comprehensive results are not expected for several more years. These agents have the potential to elicit a more robust clinical effect by the inherent associated ability to facilitate Fc-mediated macrophage clearance of mis-TTR, thereby promoting rapid amyloid degradation. This mechanism of action is distinct from that of stabilizers and silencers, which both delay the progression of ATTR-CA.

A recent indirect indication of the potential efficacy of therapeutic mAbs came from observations of natural polyclonal antibodies in patients with ATTR-CA who exhibited clinical improvement in this study of Fontana *et al*. [9, 10]. Here, we show that a previously identified therapeutic mAb, beneficial in models of ATTR cardiomyopathy [8], exhibits binding properties to mis-TTR similar to those of patient-derived anti-TTR antibodies associated with noted clinical benefits [9, 10]. This finding suggests that therapeutic mAbs ahead of expected clinical results have the potential to target patient-derived mis-TTR in ATTR-CA patients and, thus, exert a true, potent disease-modifying effect.

### Limitation

The monoclonal antibody was only tested against two polyclonal antibodies from ATTR-CA patients and thus do not necessarily reflect the entire population with anti-TTR antibodies experiencing regression.

## 5. Conclusions

Herein, we show for the first time that our previously developed monoclonal antibody targeting misfolded TTR, exhibits similar binding properties to natural polyclonal antibodies obtained from patients with ATTR-CA that experienced clinical stabilization/regression. These findings make this monoclonal antibody, an attractive candidate for future clinical testing as a potential disease modifying agent in ATTR amyloidosis.

## Data Availability

The datasets used in the current study are available from the corresponding author on reasonable request.

## Abbreviations

ATTR-CA, transthyretin cardiac amyloidosis; TTR, transthyretin.
